# Host Gene SEL1L Involved in Endoplasmic Reticulum-Associated Degradation Pathway Could Inhibit Hepatitis B Virus at RNA, DNA, and Protein Levels

**DOI:** 10.3389/fmicb.2019.02869

**Published:** 2019-12-13

**Authors:** Jinyu Wang, Jing Li, Jingwen Wu, Minhui Dong, Zhongliang Shen, Yong Lin, Fahong Li, Yongmei Zhang, Richeng Mao, Mengji Lu, Jiming Zhang

**Affiliations:** ^1^Department of Infectious Diseases, Huashan Hospital, Fudan University, Shanghai, China; ^2^Institute of Virology, University Hospital Essen, University Duisburg‐Essen, Essen, Germany; ^3^Key Laboratory of Medical Molecular Virology of the Ministry of Education (MOE) and Ministry of Health (MOH), Fudan University, Shanghai, China

**Keywords:** hepatitis B virus, SEL1L, endoplasmic reticulum-associated degradation, endoplasmic reticulum quality control-autophagy, immune tolerant, inactive carrier

## Abstract

Hepatitis B virus (HBV) belongs to the Hepadnaviridae family of enveloped DNA viruses. Recent studies have found that host factors can suppress HBV replication. HBV envelope proteins are reported to be degraded by the endoplasmic reticulum-associated degradation (ERAD) pathway. As a component of the ERAD pathway, suppressor of lin-12-like 1 (SEL1L) was earlier found to be upregulated in the inactive carrier phase of chronic HBV infection relative to that in the immune tolerant phase. However, the role of SEL1L in regulating HBV replication remains largely unknown. In this study, we found the levels of HBV RNA, DNA, and core and envelope proteins to be significantly downregulated by SEL1L overexpression and upregulated by SEL1L silencing in Huh7 cells transiently transfected with an overlength HBV genome. Similar upregulation was observed in HepG2.2.15 cells as well. SEL1L co-localized with HBV surface antigen (HBsAg), which changed its staining pattern. Treatment with an inhibitor of ERAD pathway remarkably increased intracellular S protein. Surprisingly, silencing SEL1L to block the ERAD pathway activated an alternative ER quality control (ERQC)-autophagy pathway, which might account for the increased HBV RNAs and core protein. Together, our results demonstrate that SEL1L is a host restriction factor that exerts anti-HBV effect through ERAD and alternative ERQC-autophagy pathway.

## Introduction

Hepatitis B virus (HBV) infection is a common public health concern worldwide (Schweitzer et al., 2015). Patients with chronic hepatitis B (CHB) are at high risk of developing liver cirrhosis and hepatocellular carcinoma (Tiollais et al., 1985). The natural history of chronic HBV infection refers to four phases, which is immune tolerant (IT), immune active (IA), inactive carrier status (IC), and HBeAg-negative active chronic hepatitis (ENH; European Association for the Study of the Liver. Electronic address: easloffice@easloffice.eu and European Association for the Study of the Liver, 2017).

Although the immune tolerant (IT) and inactive carrier (IC) phases of CHB are considered to maintain a similar inactivated host immune status, they have entirely different virological characteristics. Thus, viral loads are typically very high (>10^7^ IU/ml) in IT subjects although usually <2,000 IU/ml in patients at IC phase, suggesting the existence of host immunity-independent restrictive factors against the virus. Recently, we found the intrahepatic mRNA level of host gene suppressor of lin-12-like 1 (SEL1L) to be significantly upregulated in patients at IC phase compared to that in patients at IT phase (Liu et al., 2018). SEL1L protein is a core component of an endoplasmic reticulum (ER) quality control pathway, namely the endoplasmic reticulum-associated degradation (ERAD) pathway, which in turn plays a vital role in preserving a secretory function by recognizing terminally misfolded polypeptides and processing them for proteasomal degradation (Vembar and Brodsky, 2008).

HBV is a non-cytopathic, hepatotropic virus belonging to the Hepadnaviridae family. The virion is an enveloped icosahedral nucleocapsid containing a partially double-stranded relaxed circular (RC) DNA genome of 3.2 kb. The synthesized envelope proteins of HBV-infected hepatocytes are translocated into the endoplasmic reticulum (ER), where *N*-glycosylation, folding, followed by oligomerization occurring (Gerlich et al., 1992). Several viruses were found to trigger the ERAD pathway (Isler et al., 2005; Yu et al., 2006; Barry et al., 2010). A previous report had found HBV to activate the ERAD pathway by increased expression of ER degradation-enhancing mannosidase-like proteins (EDEMs), thus leading to reduced amount of intracellular envelope proteins (Lazar et al., 2012). However, whether SEL1L, a vital component of ERAD pathway, can regulate HBV life cycle, still remains unknown.

In the present study, we demonstrated that SEL1L functioned as a host restriction factor against HBV replication. Interestingly, silencing SEL1L could activate ER quality control (ERQC)-autophagy pathway, which was considered as an alternative route for protein degradation.

## Materials and Methods

### Cell Culture and Transfection

Human hepatoma cell line Huh7 was maintained in DMEM medium (Gibco, USA), supplemented with 10% fetal bovine serum, 10 mM HEPES, 100 U/ml penicillin, and 100 μg/ml streptomycin. Cells were seeded in 12-well plates at a density of 1.0 × 10^6^ cells per well. Each well was transfected with a total of 3 μg of plasmid using Lipofectamine 3000 (Invitrogen, USA) following the manufacturer’s instruction. The human hepatoma cell line HepG2.2.15 harboring the integrated dimer of HBV genome (GenBank accession number: U95551) was cultured using the same medium, except for supplementation with 400 μg/ml of G418 (Gibco, USA).

### Plasmids and Reagents

HBV replication competent plasmid pHBV1.3 (genotype D, subtype ayw, GenBank accession number V01460.1), in which the transcription of viral pregenomic RNA (pgRNA) is governed by an authentic HBV core promoter, was constructed as described previously (Mao et al., 2011). Plasmids SEL1L-FLAG and SEL1L-GFP were synthesized by GeneChem Co. (China). Plasmid HBsAg-mCherry was given as a gift from Prof. Xinwen Chen, Wuhan, China. Plasmid HBs-2-s, expressing low level of HBsAg, was kindly provided by Dr. Reinhold Schirmbeck and Dr. William Carman.

The siRNA designed to silence SEL1L expression (siSEL1L, cat.no. SI02664494) and negative control siRNA (siNC, cat.no. 1022076) was purchased from Qiagen (Germany). Kifunensine (KIF, K1140), 3-methyladenine (3-MA, M928), and chloroquine (CQ, C6628) were purchased from Sigma-Aldrich (USA).

### Analysis of Hepatitis B Virus DNA and RNA

Intracellular HBV core DNA and total RNA were extracted as described previously (Mao et al., 2011). For DNA analysis, HBV core DNA was separated by electrophoresis on a 1.5% agarose gel and transferred onto Hybond-XL membrane (GE Healthcare). For RNA analysis, 10 μg of total cellular RNA was resolved in a 1.5% agarose gel containing 2.2 M formaldehyde and transferred onto a Hybond-N+ membrane (GE Healthcare). Membranes were probed with either α-^32^P-UTP-labeled minus or plus strand-specific full-length HBV riboprobe and then exposed to a phosphor imager screen. Hybridization signals were measured with a computerized imaging system (ImageJ software).

### Western Blot Assay

Cells were seeded in 12-well plates and washed twice with PBS followed by lysing in 200 μl of Red Loading Buffer (Cell Signaling Technology). Ten microliters of the cell lysate were then resolved by electrophoresis in 12% SDS-PAGE, and proteins were transferred onto nitrocellulose filter membrane (GE Healthcare, USA). The membranes were blocked with 5% skim milk and incubated with antibodies against SEL1L (ab78298, Abcam, UK), EDEM1 (ab209660, Abcam, UK), HBsAg (ab9193, Abcam, UK), HBcAg (ab8639, Abcam, UK), LC3 (Cell Signaling Technology, USA), p62 (Cell Signaling Technology, USA), or β-actin (Cell Signaling Technology, USA). The membranes were washed with 1× TBST (as appropriate) and incubated with secondary antibodies (315-035-048, 111-035-045, Jackson ImmunoResearch, USA). Immunoreactive bands were captured by enhanced chemiluminescence system (RPN2106, GE Healthcare, USA).

### Luciferase Reporter Assay

The Dual-Glo luciferase reporter assay system (E2940, Promega, USA) was used to detect both firefly and *Renilla* (as internal control) luciferase activity. The firefly luciferase reporter plasmids pSP1, pSP2, pCP, and pXP (containing HBV promoters) were generated and used as previously described (Zhang et al., 2011).

### Immunofluorescence

Huh7 cells were seeded on cover slips and transfected with plasmids or siRNAs. Forty-eight hours after transfection, cells were fixed in 4% paraformaldehyde for 10 min and permeabilized with 0.1% Triton X-100 for 10 min. Nuclei were stained with DAPI. HBsAg and LC3 were stained with Anti-Hepatitis B Virus Surface Antigen (Ad/Ay) antibody (ab9193, Abcam, UK) and LC3B (D11) XP^®^ Rabbit mAb (3,868, Cell Signaling Technology, USA). Co-localization of SEL1L or LC3 (green) with HBsAg (red) was determined using a confocal microscope (LSM 710; Carl Zeiss) with objectives Plan-Apochromat 63×/1.40 oil Iris M27. Images were visualized by ZEN acquisition software (2012; Carl Zeiss) and analyzed by ImageJ.

### Histological Analysis and Immunohistochemistry Staining

Pieces of liver tissues from patients at IT and IC phases were fixed in 10% (vol/vol) neutralized formalin. Pathological examination of tissue section was performed by a collaborating pathologist in our hospital. For immunohistochemistry staining (IHC), paraffin-embedded liver tissues were rehydrated, boiled in 1 mM EDTA for antigen retrieval, and stained with DAB substrate from Invitrogen. After incubation with SEL1L antibody (Abcam, 1:200), IHC sections were scanned using the Aperio Scanscope, and pictures were acquired at various magnifications.

### Statistical Analysis

We undertook two-way ANOVA, including multiple comparisons, using GraphPad Prism 5 (GraphPad Software, Inc., San Diego, CA), and specifying *p* < 0.05 as the standard for statistical significance. Compared and other means are shown ± standard error of the mean. All experiments were replicated three or more times.

## Results

### Intrahepatic SEL1L Expression Was Significantly Higher in Inactive Carrier Subjects Than in Immune Tolerant Ones

**Figure 1:**
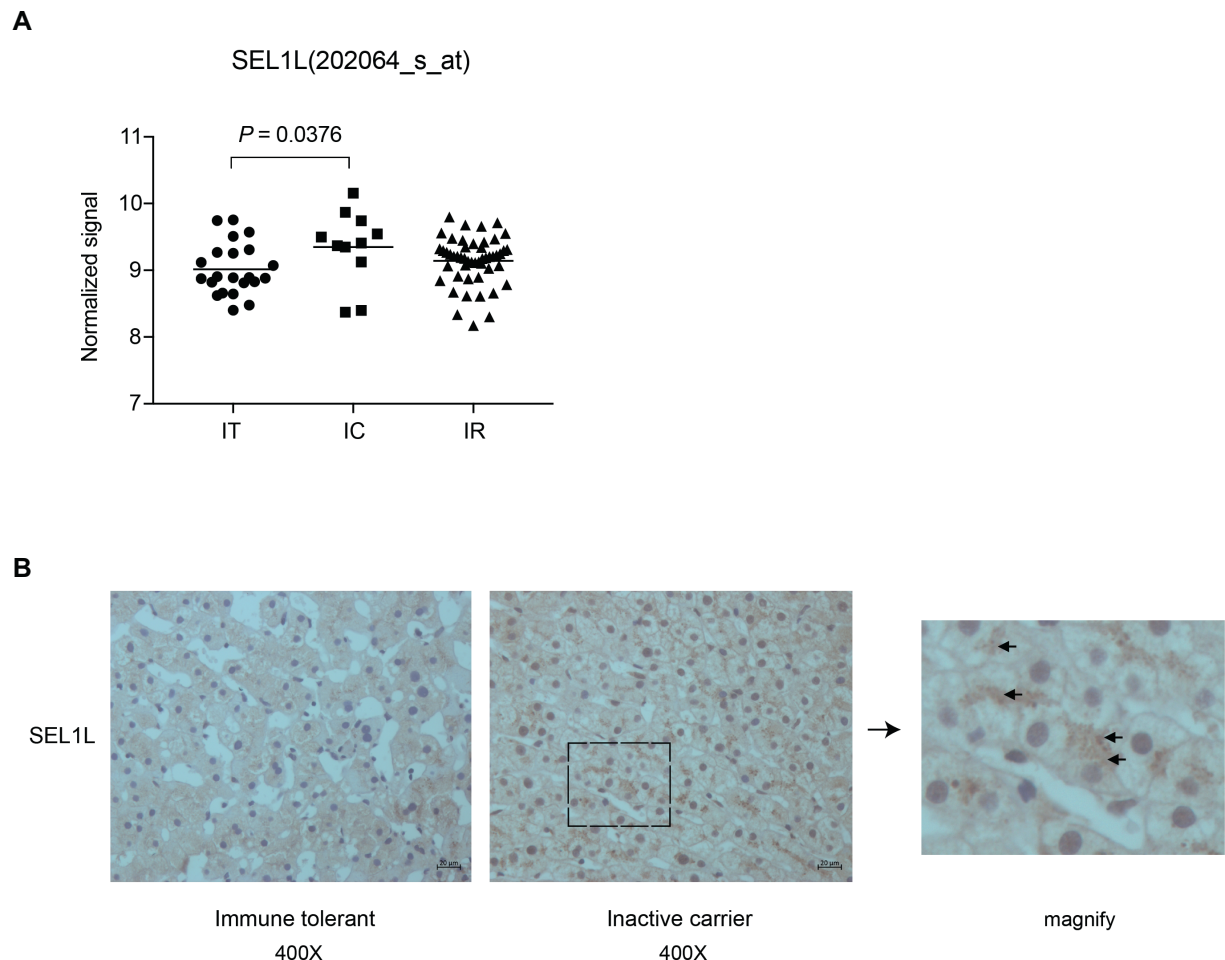
Intrahepatic expression of SEL1L was significantly higher in inactive carrier patients than in immune tolerant patients. **(A)** Liver biopsies were performed in the patients and then subjected to RNA extraction and microarray analysis using Affymetrix Human Genome U133 Plus 2.0 Array in our previous studies (Liu et al., 2018). The intrahepatic SEL1L expression was displayed in scatter plots. The mean expression levels of SEL1L in the immune tolerant (IT), inactive carrier state (IC), and immune reactive (IR) patients were calculated and compared. *p* < 0.05 was considered significant. **(B)** Two sheets of liver tissues from immune tolerant and inactive carrier patients, respectively, were fixed and stained with SEL1L antibody (yellow).

Liver biopsies of 83 treatment-naïve patients, from four natural-history phases, were followed by subsequent RNA extraction and microarray analysis. Supplementary Table 1 contains an overview of the CHB patients’ clinical and virological characteristics. Patients in IT and IC phases had different HBV DNA loads, with normal alanine aminotransferase (ALT) levels. Our previous study had revealed a set of host genes, including SEL1L, which may be involved in the control of HBV replication in IC phase (Liu et al., 2018). As shown in Figure 1A, SEL1L expression was significantly higher in IC phase than in IT phase. Next, we investigated SEL1L distribution *in vivo*. Liver tissues from treatment-naïve patients with chronic hepatitis B were fixed and stained with SEL1L antibody. For patients in IT phase, representative images showed SEL1L to be mostly distributed homogeneously. However, for patients in IC phase, SEL1L accumulated as visible coarse granules, which suggested the possibility of SEL1L protein to aggregate and form a functional complex (Figure 1B).

### Hepatitis B Virus RNA, DNA, and Core and Envelope Proteins Were Increased by SEL1L Silencing and Decreased by Its Overexpression in Human Hepatoma Cells

**Figure 2:**
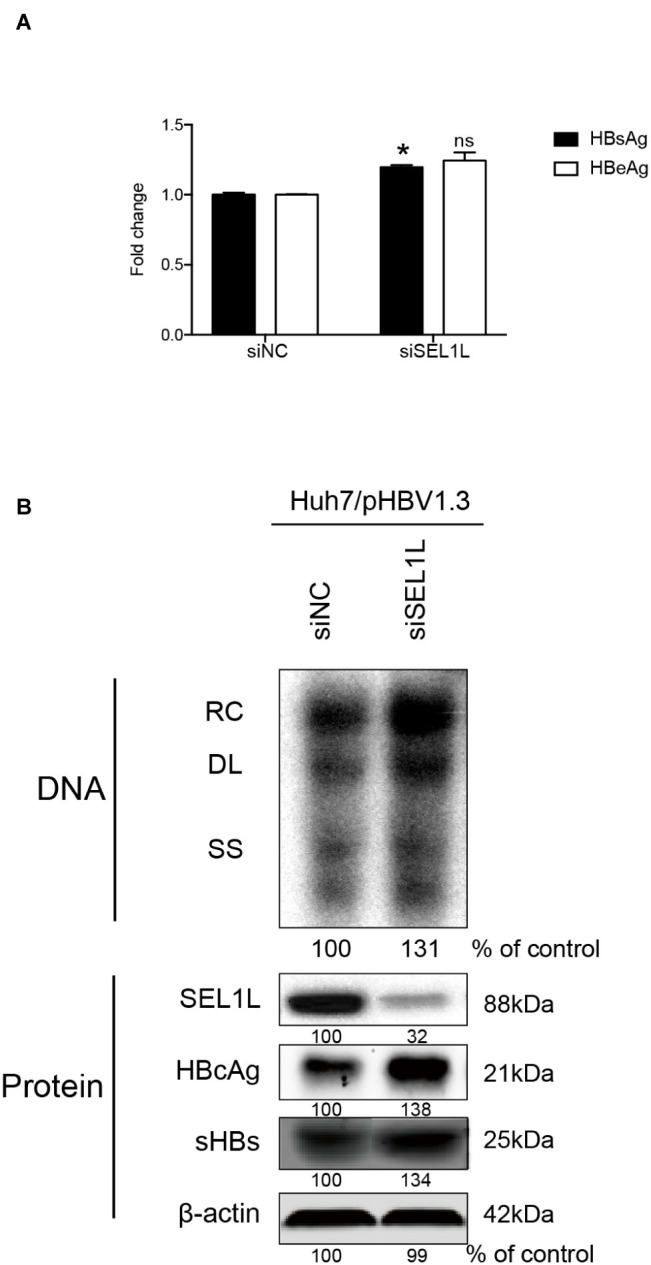
Knocking down of SEL1L increased HBV replication in Huh7 cells. **(A)** Huh7 cells in 12-well plates were co-transfected with 1.5 μg pHBV 1.3 containing 1.3 mer genome-length HBV sequences and 20 nM control siRNA or siSEL1L and harvested after 72 h. Secreted HBsAg and HBeAg from the supernatant of cell culture were measured and reported as fold change. **(B)** Core DNA and proteins were analyzed by southern blot (upper panels) and western blot (lower panels) assays, respectively. The positions of relaxed circular (RC), single-stranded (SS) DNA were indicated. The expression of intracellular viral proteins including surface and core proteins was also measured. The levels of β-actin served as a loading control. The relative gray-scale value for each lane of DNA and proteins was measured by Image J software as the percentage of DNA in control cells. ^*^p < 0.05; ns, not significant.

**Figure 3:**
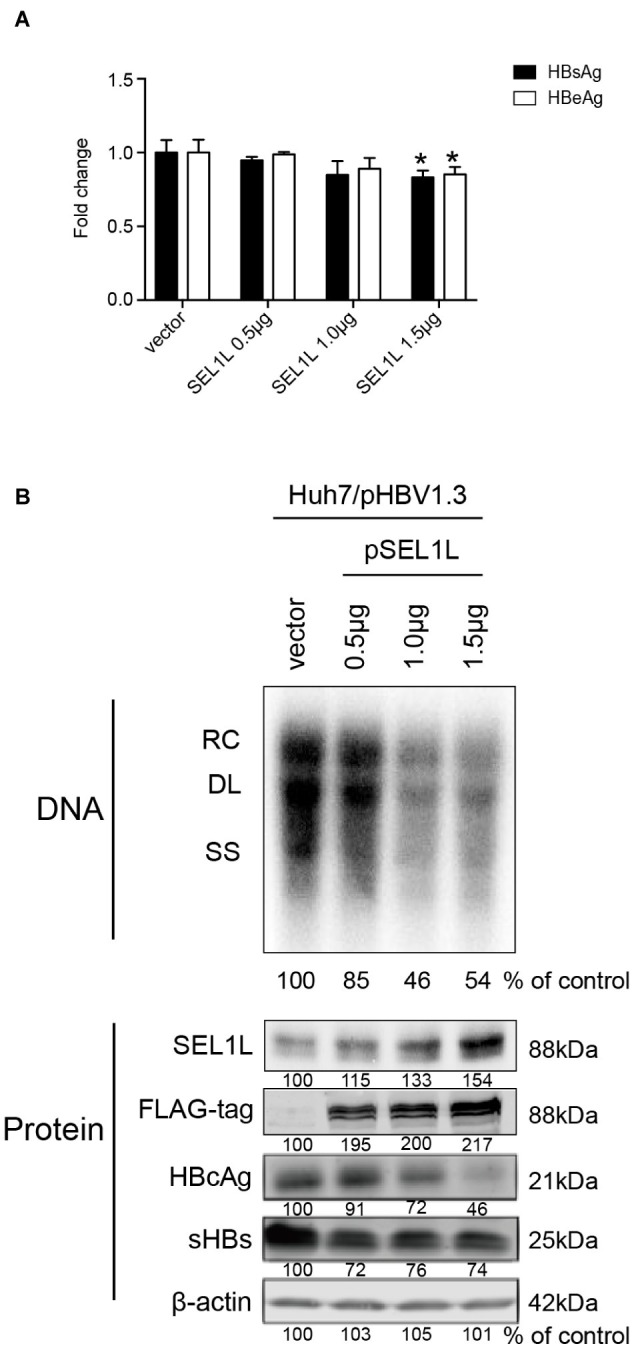
Overexpression of SEL1L decreased HBV replication in Huh7 cells. Huh7 cells in 12-well plates were co-transfected with 1.5 μg pHBV1.3 and control empty vector or constructed plasmid expressing flag-tagged pSEL1L in a dose-dependent manner. Secreted viral proteins **(A)**, HBV DNA, and proteins **(B)** were detected as described above. The expression of SEL1L was revealed by western blot analysis with FLAG antibodies. ^*^p < 0.05.

Huh7 cells were transiently transfected with a 1.3-mer construct of the HBV genome, together with SEL1L siRNA, thereby increasing HBV DNA levels relative to that in co-transfection with control siRNA. Reduced expression of SEL1L was confirmed by western blot (Figure 2B, bottom panels) with no cytotoxic effect observed. Knockdown of SEL1L increased the secreted HBsAg (Figure 2A, *p* = 0.0142) and HBeAg (*p* = 0.1331) in the supernatant compared to control group and generated higher levels of HBV DNA (Figure 2B, top panels) as well as intracellular core and S proteins (Figure 2B, bottom panels). Similar results were obtained in HepG2.2.15 cells (Supplementary Figure 1A) as well. Conversely, co-transfection with a 1.3-mer construct of the HBV genome, together with empty vector or increasing concentrations of an expression construct for human SEL1L, reduced the secreted HBV proteins (Figure 3A, HBsAg, SEL1L 1.5 μg and vector, *p* = 0.0463; HBeAg, SEL1L 1.5 μg and vector, *p* = 0.0477) and intracellular levels of replicative HBV DNA in a dose-dependent manner (Figure 3B, top panels). Similar tendencies were observed for intracellular levels of S and core proteins as well (Figure 3B, bottom panels). Secreted HBsAg from HBs-2-s could be reduced by more than 50% by SEL1L overexpression (Supplementary Figure 1B), which indicated a limited ability of SEL1L to reduce HBsAg. HBV genotypes B and C responded similarly, with overexpressed SEL1L resulting in reduced replication (Supplementary Figures 2B,D), while reduced SEL1L increased HBV DNA and secreted HBsAg (Supplementary Figures 2A,C). Collectively, these results suggested that host SEL1L protein negatively regulates HBV RNA, DNA, and proteins.

### SEL1L Could Regulate Hepatitis B Virus Replication *via* a Post-transcriptional Mechanism

**Figure 4:**
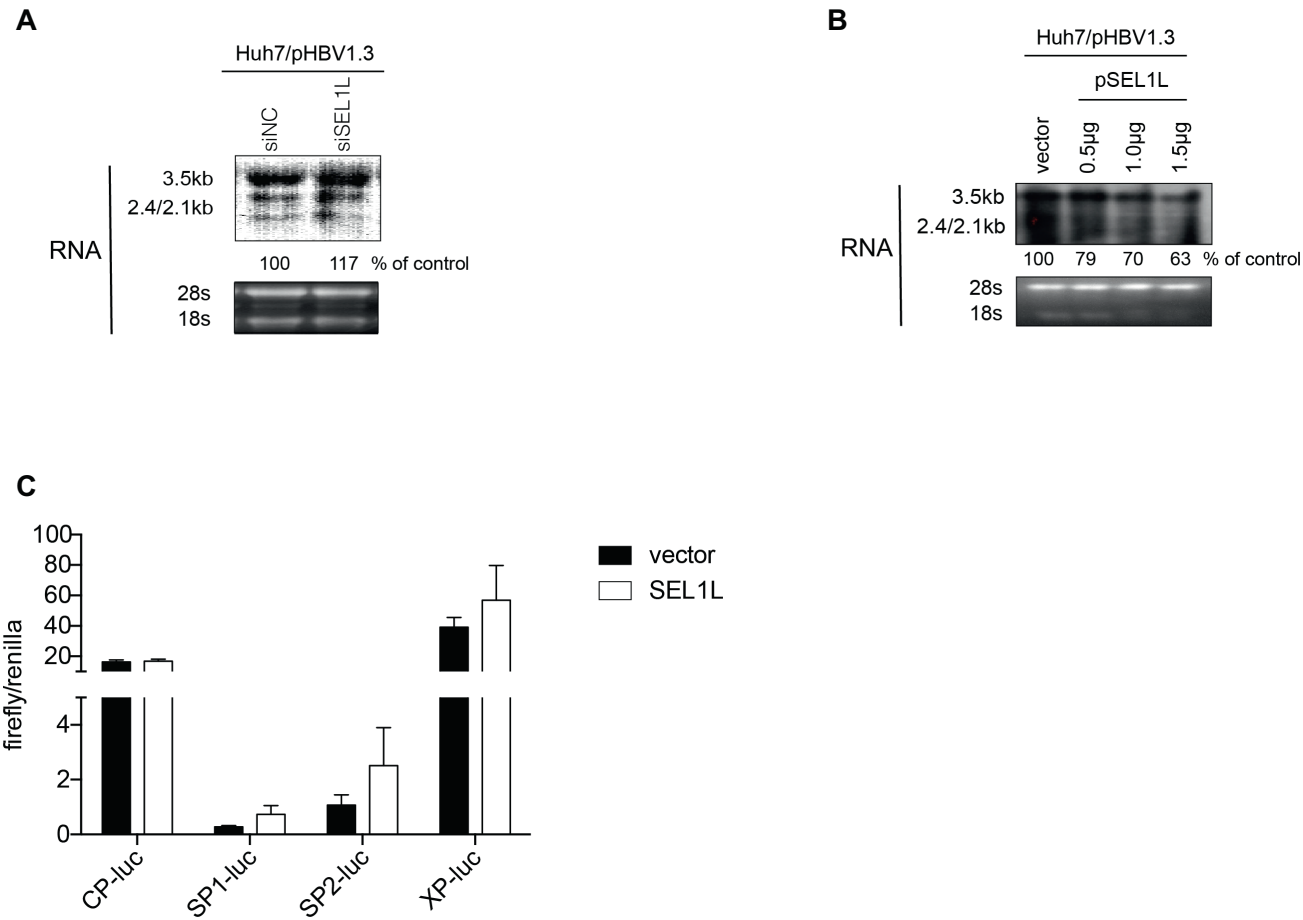
SEL1L had no significant impact on HBV RNA and promoter activities. **(A,B)** Huh7 cells in 12-well plates were co-transfected with 1.5 μg pHBV1.3 and control empty vector or constructed plasmid expressing flag-tagged pSEL1L in a dose-dependent manner, or with 20 nM control siRNA or siSEL1L and harvested after 72 h. HBV RNAs were analyzed by northern blot assay. Ribosomal RNAs (28S and 18S) were presented as loading controls. The positions of HBV 3.5, 2.4, and 2.1 kb RNAs were indicated. The relative gray-scale value for each lane of RNA was measured by Image J software as the percentage of RNA in control cells. **(C)** Huh7 cells in 24-well plates were co co-transfected with 100 ng of four HBV promoters Cp-Luc, SP1-Luc, SP2-Luc, or XP-Luc, and 100 ng of *Renilla*, plus 300 ng of control vector or plasmid SEL1L. Forty-eight hours after transfection, cells were harvested, and luciferase activity was measured.

In order to clarify whether SEL1L-mediated downregulation of HBV RNA may be attributed to a transcriptional or post-transcriptional mechanism, HBV RNA analysis and promoter reporter assays were performed. Knockdown of SEL1L did not significantly alter HBV RNAs, including the 3.5-kb pregenomic (pg) RNA, the precursor for HBV DNA replication, and subgenomic RNAs of 2.4/2.1 kb for HBV envelope protein translation (Figure 4A). SEL1L overexpression decreased HBV RNA levels slightly (Figure 4B). Moreover, SEL1L did not significantly inhibit the activities of HBV core promoter or X promoter. It, in fact, increased the activities of SP1 and SP2 promoters, although the difference did not reach statistical significance (Figure 4C).

The decay kinetics of HBV RNA were measured with and without SEL1L overexpression. HepDES19 cells were transfected with control vector or plasmid SEL1L without tetracycline and incubated for 36 h to induce HBV RNA transcription. Tetracycline was then added so that *de novo* transcription of HBV pgRNA from the transgene ceased (Supplementary Figure 3A). Time-course measurements of the decay kinetics of HBV RNA were made following cessation of expression (Supplementary Figure 3B). HBV RNA degraded no faster in HepDES19 cells with overexpressed SEL1L than that in the controls. Thus, we concluded that SEL1L did not promote such degradation and therefore had no significant effect on the stability of HBV RNA. SEL1L-mediated HBV RNA reduction was not due to transcriptional inhibition but most likely attributed to a post-transcriptional mechanism.

### Blocking the Endoplasmic Reticulum-Associated Degradation Pathway Increased Intracellular and Secreted Levels of Hepatitis B Virus Envelope Proteins

**Figure 5:**
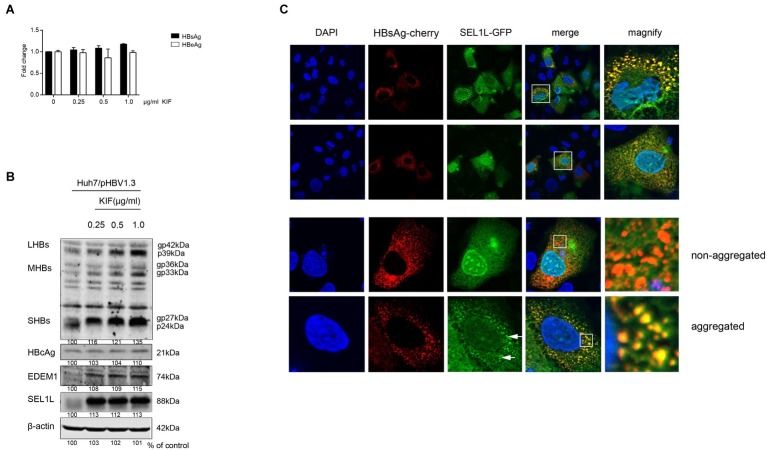
SEL1L decreased HBV envelope proteins through the ERAD pathway and may be aggregated to form granules. **(A,B)** Huh7 cells in 12-well plates were transfected with 1.5 μg pHBV1.3, followed by treating cells with ER mannosidase inhibitor KIF in a dose-dependent manner for 48 h post transfection. The expression levels of intracellular and secreted viral proteins as well as SEL1L and EDEM1 were measured as previously described. **(C)** Huh7 cells were co-transfected with 1.5 μg pSEL1L-GFP (green) and 1.5 μg HBsAg-cherry (red). The cells were fixed and stained with DAPI (blue). The colocalization of SEL1L and HBsAg was measured by confocal microscopy.

SEL1L is an adaptor protein for the ubiquitin ligase HRD1 in ERAD pathway. A previous study had demonstrated the activation of ERAD pathway by HBV, which in turn reduced the levels of three envelope proteins (Lazar et al., 2012). In the present study, we treated HBV-transfected cells with kifunensine (KIF), a potent inhibitor of ER mannosidase to block the ERAD pathway. It increased the secreted HBsAg levels in the supernatant of cultured cells in a dose-dependent manner (Figure 5A). Similar trend was observed for intracellular HBV envelope proteins (Figure 5B). Moreover, two important components of ERAD pathway, SEL1L and EDEM1, were also increased (Figure 5B), which probably resulted from the less consumption after ERAD inhibition. However, KIF did not increase HBV core protein level (Figure 5B), indicating that the decrease of core protein by SEL1L overexpression was not *via* ERAD pathway.

### SEL1L Co-localized With HBsAg in the Cytoplasm to Generate a Granular Distribution

To check the interaction of SEL1L with HBV envelope proteins, we first examined its intracellular distribution in the absence of HBV. Immunofluorescence staining showed cytoplasmic localization of SEL1L protein (Supplementary Figure 4A), with homogeneous distribution. Strikingly, when HBsAg was also expressed in Huh7 cells, SEL1L accumulated into visible coarse granules (green spots) co-localizing with HBsAg-cherry (red spots) as obvious granules (yellow spots; Figure 5C). Nevertheless, the pattern of homogeneous distribution could still be observed in some sections, despite HBsAg co-expression (Figure 5C, bottom panels). The separate localizations of SEL1L-GFP and HBsAg-cherry were shown in Supplementary Figures 4B,C. We further noted that SEL1L maintained a homogeneous, non-granular distribution where HBcAg expression was occurring in Huh7 cells (Supplementary Figure 4D). Incomplete co-localization was observed for SEL1L and HBcAg. The results suggested the association of SEL1L with HBsAg, which might be an intermediate in HBsAg degradation. Another mode is suspected as the explanation for the decrease of HBcAg when SEL1L was expressed.

### SEL1L Silencing May Promote Hepatitis B Virus Replication Through the Activation of Endoplasmic Reticulum Quality Control-Autophagy Pathway

ERAD is not the only pathway for degradation of misfolded proteins. Buchberger had demonstrated another ER quality control-autophagy (ERQC-autophagy) pathway that allows the entry of ERAD-resistant conformers, followed by degradation through the autophagosome-lysosome format (Buchberger, 2014; Houck et al., 2014). However, whether ERQC-autophagy is a compensatory pathway or parallel option for proteins that cannot be recognized, ubiquitinated, or transported by ERAD pathway remains to be clarified.

**Figure 6:**
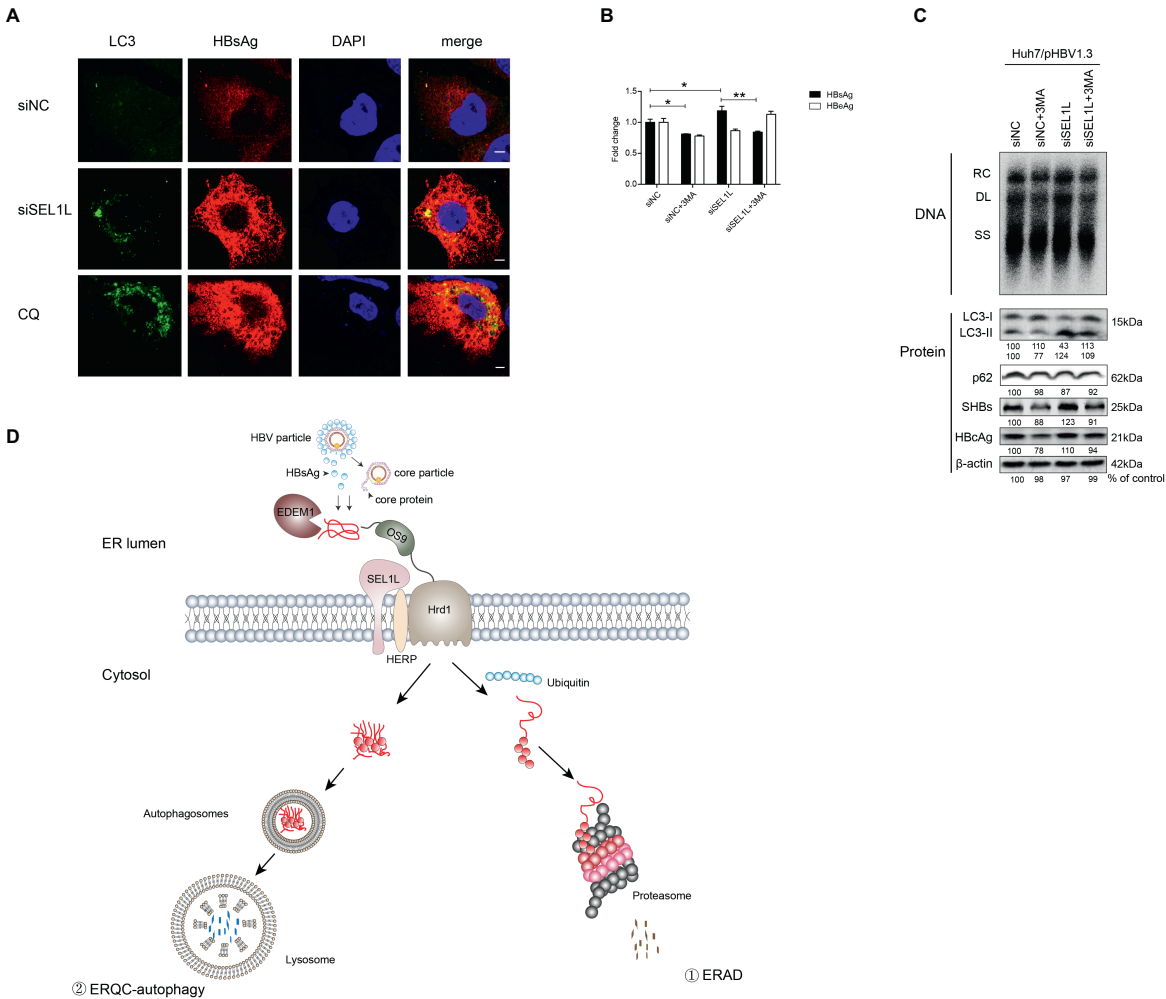
Silencing of SEL1L induced ERQC-autophagy activation. **(A)** Huh7 cells were co-transfected with 1.5 μg pHBV 1.3 and 20 nM control siRNA or siSEL1L. After 48 h, wells were fixed and incubated with primary anti-LC3, followed by staining with Alexa Fluor 488-conjugated anti-rabbit secondary antibody IgG. As for HBsAg staining, primary anti-HBsAg and Alexa Fluor 594-conjugated anti-rabbit secondary antibody IgG were used. The cells were treated with 10 μmol/L CQ for 24 h as a positive control. The images were captured by confocal microscopy. **(B,C)** Huh7 cells in 12-well plates were co-transfected with 1.5 μg pHBV 1.3 and 20 nM control siRNA or siSEL1L. A 3-MA (10 mM) was treated for 24 h before harvest. Secreted HBsAg and HBeAg from the supernatant of cell culture were measured. Analysis of LC3, p62, intracellular HBsAg, and HBcAg expression was performed by western blotting. **(D)** Degradation pathways for proteins at the endoplasmic reticulum. EDEM1 and OS-9 facilitate delivery of misfolded polypeptides to the adaptor proteins SEL1L and the HRD1 dislocation machinery. As for HBV, envelope and core proteins proceed to the ER membrane. Misfolded proteins dislocated across the ER membrane, which are polyubiquitylated, and degraded by cytosolic proteasomes (route 1). Under conditions that limit proteasomal capacity, the dislocated substrates aggregate in the cytoplasm and accumulate in aggresomes, which are then transferred to the lysosome for selective degradation by autophagy (route 2). After fusion of the autophagosome with lysosome, ERAD-resistant protein aggregates are engulfed by autophagosomes and degraded by lysosomal hydrolases (route 2). ^*^p < 0.05; ^**^p < 0.01.

To examine the effect of SEL1L knockdown on autophagy, plasmid pHBV1.3 and siRNAs were co-transfected into Huh7 cells. As shown in Figure 6A, knockdown of SEL1L increased LC3 and HBsAg expression, which was observed to be co-localized. CQ, which is an autophagy activator, was used as a positive control. It, therefore, suggests that knockdown of SEL1L induced autophagy activation.

As described above, SEL1L overexpression decreased HBV RNA and core protein, which cannot be otherwise explained by the ERAD pathway. Hence, we added 3-methyladenine (3-MA), an autophagy inhibitor, to the culture medium, either alone or together with the knockdown of SEL1L, in Huh7 cells transfected with pHBV 1.3 plasmid. Results showed that HBV core DNA secreted and intracellular HBsAg and HBeAg levels were reduced by 3-MA treatment, as was the level of LC3-II (Figures 6B,C), thus suggesting a reduction in autophagy (consistent with previous studies; Lin et al., 2018). Interestingly, knockdown of SEL1L had the opposite effect and led to a significant increase in LC3-II. If 3-MA treatment was performed along with knockdown of SEL1L, LC3-II levels were still higher than 3-MA treatment alone but slightly lower than in SEL1L knockdown alone (Figure 6C). HBV DNA and core protein have been earlier reported to be increased by autophagy (Liu et al., 2014; Lin et al., 2017). Thus, we suspect that knockdown of SEL1L blocked the ERAD pathway due to deficiency of a key component. The alternative ERQC-autophagy is then activated as a compensatory mechanism for degradation of conformers (Figure 6D). As for HBV, ERQC-autophagy activation may increase both HBV RNA and core protein levels.

## Discussion

Our present study raised the possibility of SEL1L playing an indispensable role in either ERAD or ERQC-autophagy pathway to degrade viral proteins and inhibit HBV replication. Overexpression of SEL1L resulted in the reduction of HBV DNA, RNA, and viral proteins, whereas knockdown of SEL1L showed opposite results. We noticed that SEL1L did not inhibit HBV promoters, which suggested post-transcriptional mechanisms to be involved in the reduction of HBV RNA and core proteins. Furthermore, our data suggested that while SEL1L probably decreased HBV envelope proteins through the ERAD pathway, SEL1L silencing increased HBV RNAs and core protein possibly *via* the ERQC-autophagy pathway.

SEL1L is a highly conserved ER-resident trans-membrane protein involved in the degradation of a subset of misfolded ER proteins through ERAD pathway [13,14,17]. SEL1L was found to be indispensable for mammalian ERAD and ER homeostasis (Sun et al., 2014). However, the relationship between SEL1L and HBV infection had not been investigated earlier. Infection of several viruses has been shown to induce unfolded protein response (UPR; Ambrose and Mackenzie, 2011; Trujillo-Alonso et al., 2011). Recently, importance of the ERAD pathway in modulating HCV and HBV infection was demonstrated with more attention on EDEMs. EDEMs interact with HCV glycoproteins, inducing increased ubiquitylation (Saeed et al., 2011). As for HBV, ERAD pathway was found to reduce the intracellular level of envelope proteins (Lazar et al., 2012). The downstream SEL1L is usually thought to form a complex with HRD1 and OS9, and control HRD1 stability (Cattaneo et al., 2008). Our data demonstrated the decrease of HBV envelope proteins by SEL1L overexpression, thus resulting in ERAD pathway activation; however, no effect was observed on HBV core protein when ERAD was blocked by kifunensine. Besides, silencing OS9 and HRD1 did not alter HBsAg or core protein levels (data not shown). We, therefore, suspected that SEL1L may occupy an important site in another pathway for the reduction of core protein.

SEL1L may function as HRD1-independent pattern in cells [15]. A recent study showed that a SEL1L-LC3-I complex was formed to deliver ERAD-associated proteins, which controlled the efficacy of ERAD (Noack et al., 2014). Sharma et al. found replication of Japanese encephalitis virus to be suppressed by autophagy occurring on LC3-I-containing membranes, which are activated by silencing of SEL1L and EDEM1 (Sharma et al., 2014); this suggested that SEL1L deficiency activates autophagy. On the contrary, Sun et al. proposed that SEL1L deficiency did not induce increase of autophagy in the pancreas, as evidenced by LC3B cleavage in mice (Sun et al., 2014).

Autophagy is known to impact on HBV replication significantly (Sir et al., 2010; Li et al., 2011; Liu et al., 2014). Independent studies have consistently demonstrated that autophagy inhibition strongly decreases HBV replication in hepatic cells (Sir et al., 2010; Li et al., 2011). Additionally, autophagy deficiency was found to strongly reduce HBV replication in a transgenic mouse model (Tian et al., 2011). In fact, at the late stage of autophagy, HBV replication is enhanced (Lin et al., 2018). In yeast, autophagy function is a distinct degradation pathway to remove proteins from the ER (Kruse et al., 2006). Relation between ERAD and autophagy was established when Houck et al. reported that novel ERQC-autophagy could degrade ER-resistant proteins (Houck et al., 2014). The key structure of polypeptide substrate, which can be recognized by ERQC-autophagy, is yet to be defined, and the relationship between ERAD and ERQC-autophagy remains largely unknown. It is, however, tempting to suggest that in case of insufficient degradation by ERAD, usually due to abnormally structured polypeptides or deficiency of ERAD components, proteins aggregate on the ER membrane to increase ER stress and stimulate autophagy. Our data suggested that SEL1L deficiency is actually related to the increase of autophagy in liver, suggesting the possibility of organ-specific properties of ERQC-autophagy. Due to the differential structures of HBV core protein and surface proteins, they might enter different degradation pathways induced by SEL1L overexpression. However, we did not clarify the details of this event, which deserves further investigation. The demonstration of host-virus interaction will be definitely helpful in exploring new methods for therapy of chronic hepatitis B.

In summary, our study suggested SEL1L as indispensable in ERAD as well as alternative ERQC-autophagy pathway for degradation of HBV proteins and control of HBV replication. Although the exact mechanisms for degradation of HBV proteins remain unclear at the moment, the novel function of SEL1L is worth further investigation.

## Data Availability Statement

All datasets generated for this study are included in the article/Supplementary Material.

## Ethics Statement

The studies involving human participants were reviewed and approved by the ethics committee of Fudan University affiliated Hushan Hospital. The patients/participants provided their written informed consent to participate in this study.

## Author Contributions

JWa conceived the project. JWa and JL wrote the manuscript. JWa, JL, JWu MD, ZS, and YL conducted the experiments. FL, YZ, RM, ML, and JZ offered the helpful guidance of this study.

### Conflict of Interest

The authors declare that the research was conducted in the absence of any commercial or financial relationships that could be construed as a potential conflict of interest.

## References

[ref1] Ambrose R. L. Mackenzie J. M. (2011). West Nile virus differentially modulates the unfolded protein response to facilitate replication and immune evasion. J. Virol. 85, 2723–2732. 10.1128/JVI.02050-10, PMID: 21191014 PMC3067947

[ref2] Barry G. Fragkoudis R. Ferguson M. C. Lulla A. Merits A. Kohl A.. (2010). Semliki forest virus-induced endoplasmic reticulum stress accelerates apoptotic death of mammalian cells. J. Virol. 84, 7369–7377. 10.1128/JVI.02310-09, PMID: 20427528 PMC2898233

[ref3] Buchberger A. (2014). ERQC autophagy: yet another way to die. Mol. Cell 54, 3–4. 10.1016/j.molcel.2014.03.037, PMID: 24725593

[ref4] Cattaneo M. Otsu M. Fagioli C. Martino S. Lotti L. V. Sitia R.. (2008). SEL1L and HRD1 are involved in the degradation of unassembled secretory Ig-mu chains. J. Cell. Physiol. 215, 794–802. 10.1002/jcp.21364, PMID: 18314878

[ref5] European Association for the Study of the Liver. Electronic address: easloffice@easloffice.eu and European Association for the Study of the Liver (2017). EASL 2017 Clinical Practice Guidelines on the management of hepatitis B virus infection. J. Hepatol. 67, 370–398. 10.1016/j.jhep.2017.03.021 28427875

[ref6] Gerlich W. H. Heermann K. H. Lu X. (1992). Functions of hepatitis B surface proteins. Arch. Virol. Suppl. 4, 129–132. 1450681 10.1007/978-3-7091-5633-9_28

[ref7] Houck S. A. Ren H. Y. Madden V. J. Bonner J. N. Conlin M. P. Janovick J. A.. (2014). Quality control autophagy degrades soluble ERAD-resistant conformers of the misfolded membrane protein GnRHR. Mol. Cell 54, 166–179. 10.1016/j.molcel.2014.02.025, PMID: 24685158 PMC4070183

[ref8] Isler J. A. Skalet A. H. Alwine J. C. (2005). Human cytomegalovirus infection activates and regulates the unfolded protein response. J. Virol. 79, 6890–6899. 10.1128/JVI.79.11.6890-6899.2005, PMID: 15890928 PMC1112127

[ref9] Kruse K. B. Brodsky J. L. McCracken A. A. (2006). Characterization of an ERAD gene as VPS30/ATG6 reveals two alternative and functionally distinct protein quality control pathways: one for soluble Z variant of human alpha-1 proteinase inhibitor (A1PiZ) and another for aggregates of A1PiZ. Mol. Biol. Cell 17, 203–212. 10.1091/mbc.e04-09-0779 16267277 PMC1345659

[ref10] Lazar C. Macovei A. Petrescu S. Branza-Nichita N. (2012). Activation of ERAD pathway by human hepatitis B virus modulates viral and subviral particle production. PLoS One 7:e34169. 10.1371/journal.pone.0034169, PMID: 22461906 PMC3312915

[ref11] Li J. Liu Y. Wang Z. Liu K. Wang Y. Liu J.. (2011). Subversion of cellular autophagy machinery by hepatitis B virus for viral envelopment. J. Virol. 85, 6319–6333. 10.1128/JVI.02627-10, PMID: 21507968 PMC3126540

[ref12] Lin Y. Deng W. Pang J. Kemper T. Hu J. Yin J.. (2017). The microRNA-99 family modulates hepatitis B virus replication by promoting IGF-1R/PI3K/Akt/mTOR/ULK1 signaling-induced autophagy. Cell. Microbiol. 19, 1–15. 10.1111/cmi.12709, PMID: 27886437

[ref13] Lin Y. Wu C. Wang X. Kemper T. Squire A. Gunzer M. (2018). Hepatitis B virus is degraded by autophagosome-lysosome fusion mediated by Rab7 and related components. Protein Cell 10, 60–66. 10.1007/s13238-018-0555-2 PMC6321816 29876903

[ref14] Liu B. Fang M. Hu Y. Huang B. Li N. Chang C.. (2014). Hepatitis B virus X protein inhibits autophagic degradation by impairing lysosomal maturation. Autophagy 10, 416–430. 10.4161/auto.27286, PMID: 24401568 PMC4077881

[ref15] Liu H. Li F. Zhang X. Yu J. Wang J. Jia J.. (2018). Differentially expressed intrahepatic genes contribute to control of hepatitis B virus replication in the inactive carrier phase. J. Infect. Dis. 217, 1044–1054. 10.1093/infdis/jix683, PMID: 29300924

[ref16] Mao R. Zhang J. Jiang D. Cai D. Levy J. M. Cuconati A. (2011). Indoleamine 2,3-dioxygenase mediates the antiviral effect of gamma interferon against hepatitis B virus in human hepatocyte-derived cells. J. Virol. 85, 1048–1057. 10.1128/JVI.01998-10 21084489 PMC3019998

[ref17] Noack J. Bernasconi R. Molinari M. (2014). How viruses hijack the ERAD tuning machinery. J. Virol. 88, 10272–10275. 10.1128/JVI.00801-14, PMID: 24990995 PMC4178841

[ref18] Saeed M. Suzuki R. Watanabe N. Masaki T. Tomonaga M. Muhammad A.. (2011). Role of the endoplasmic reticulum-associated degradation (ERAD) pathway in degradation of hepatitis C virus envelope proteins and production of virus particles. J. Biol. Chem. 286, 37264–37273. 10.1074/jbc.M111.259085, PMID: 21878646 PMC3199473

[ref19] Schweitzer A. Horn J. Mikolajczyk R. T. Krause G. Ott J. J. (2015). Estimations of worldwide prevalence of chronic hepatitis B virus infection: a systematic review of data published between 1965 and 2013. Lancet 386, 1546–1555. 10.1016/S0140-6736(15)61412-X, PMID: 26231459

[ref20] Sharma M. Bhattacharyya S. Nain M. Kaur M. Sood V. Gupta V.. (2014). Japanese encephalitis virus replication is negatively regulated by autophagy and occurs on LC3-I- and EDEM1-containing membranes. Autophagy 10, 1637–1651. 10.4161/auto.29455, PMID: 25046112 PMC4206540

[ref21] Sir D. Tian Y. Chen W. L. Ann D. K. Yen T. S. Ou J. H. (2010). The early autophagic pathway is activated by hepatitis B virus and required for viral DNA replication. Proc. Natl. Acad. Sci. U. S. A. 107, 4383–4388. 10.1073/pnas.0911373107 20142477 PMC2840127

[ref22] Sun S. Shi G. Han X. Francisco A. B. Ji Y. Mendonca N.. (2014). Sel1L is indispensable for mammalian endoplasmic reticulum-associated degradation, endoplasmic reticulum homeostasis, and survival. Proc. Natl. Acad. Sci. U. S. A. 111, E582–E591. 10.1073/pnas.1318114111, PMID: 24453213 PMC3918815

[ref23] Tian Y. Sir D. Kuo C. F. Ann D. K. Ou J. H. (2011). Autophagy required for hepatitis B virus replication in transgenic mice. J. Virol. 85, 13453–13456. 10.1128/JVI.06064-11, PMID: 21957292 PMC3233133

[ref24] Tiollais P. Pourcel C. Dejean A. (1985). The hepatitis B virus. Nature 317, 489–495. 10.1038/317489a0, PMID: 2995835

[ref25] Trujillo-Alonso V. Maruri-Avidal L. Arias C. F. Lopez S. (2011). Rotavirus infection induces the unfolded protein response of the cell and controls it through the nonstructural protein NSP3. J. Virol. 85, 12594–12604. 10.1128/JVI.05620-11, PMID: 21937647 PMC3209385

[ref26] Vembar S. S. Brodsky J. L. (2008). One step at a time: endoplasmic reticulum-associated degradation. Nat. Rev. Mol. Cell Biol. 9, 944–957. 10.1038/nrm2546, PMID: 19002207 PMC2654601

[ref27] Yu C. Y. Hsu Y. W. Liao C. L. Lin Y. L. (2006). Flavivirus infection activates the XBP1 pathway of the unfolded protein response to cope with endoplasmic reticulum stress. J. Virol. 80, 11868–11880. 10.1128/JVI.00879-06, PMID: 16987981 PMC1642612

[ref28] Zhang X. Zhang E. Ma Z. Pei R. Jiang M. Schlaak J. F.. (2011). Modulation of hepatitis B virus replication and hepatocyte differentiation by microRNA-1. Hepatology 53, 1476–1485. 10.1002/hep.24195, PMID: 21520166

