# Supplementary material for: Host Gene SEL1L Involved in Endoplasmic Reticulum-Associated Degradation Pathway Could Inhibit Hepatitis B Virus at RNA, DNA, and Protein Levels
Source: Front Microbiol. 2019 Dec 13;10:2869. doi: 10.3389/fmicb.2019.02869 (PMC6923250; doi:10.3389/fmicb.2019.02869)
Supplement: Supplementary file 1 [file Data_Sheet_1.docx]

Supplementary Material

# Supplementary Figures

## Supplementary Figure 1
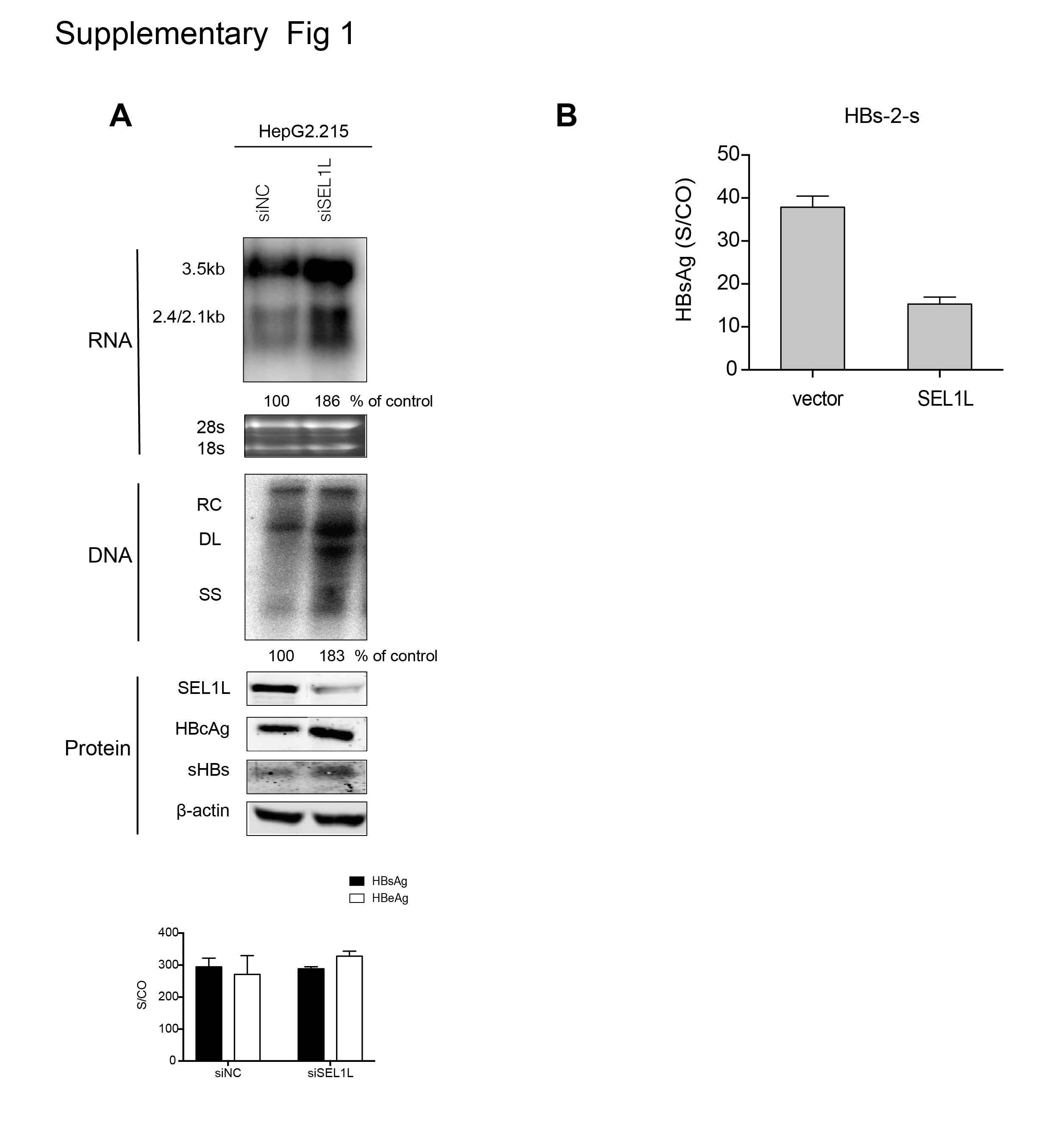


**Supplementary Figure 1.** (A) HepG2.2.15 cells in 12-well plates were transfected with 20nM control siRNA or siSEL1L and harvested after 72h. HBV RNA, DNA, intracellular and secreted viral proteins were detected as descried above. (B) Huh7 cells in 12-well plates were co-transfected with plasmids HBs-2-s and vector or pSEL1L. HBsAg levels in the supernatant were detected.

**1.2 Supplementary Figure 2**


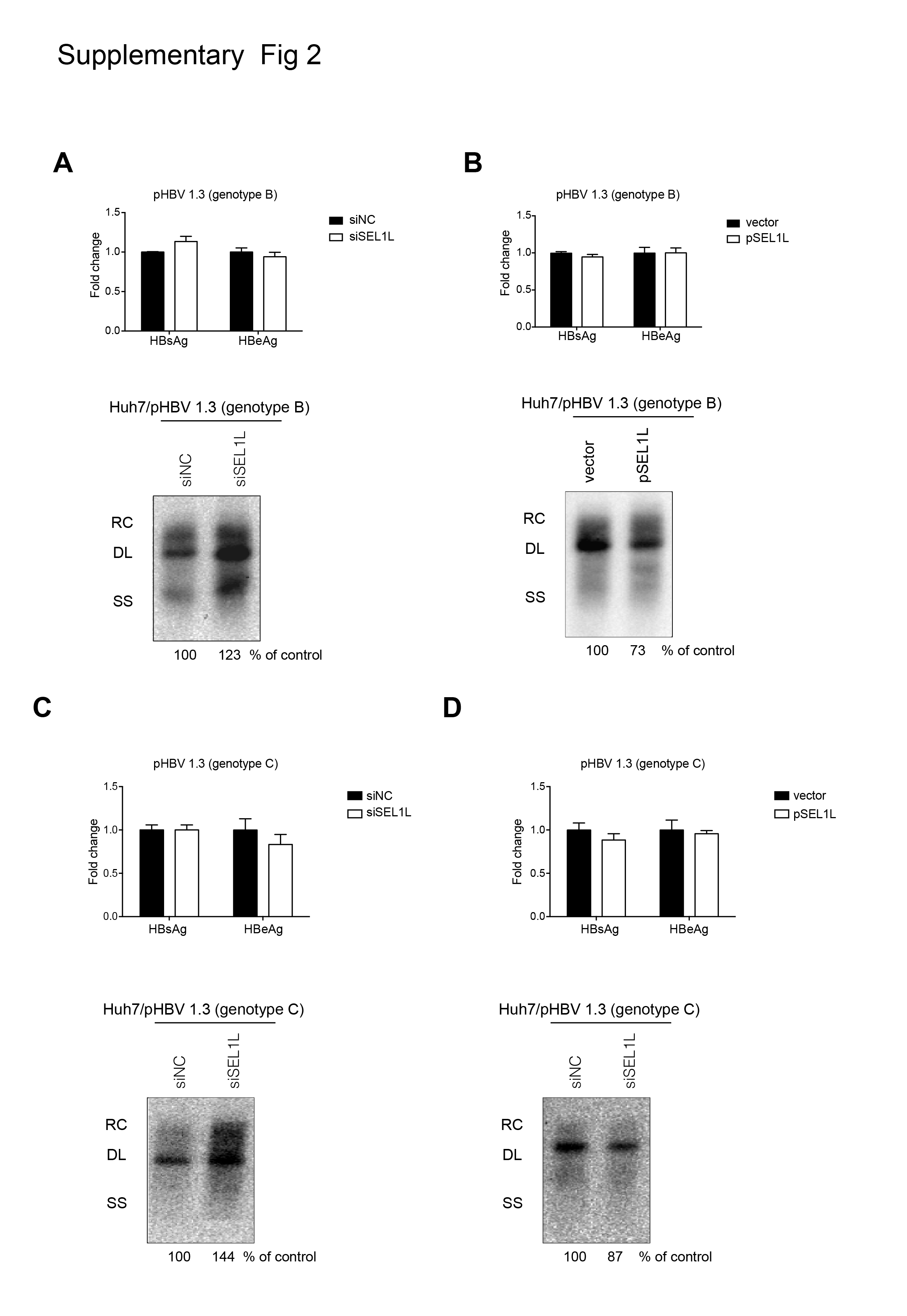


**Supplementary Figure 2.** (A and C) Huh7 cells in 12-well plates were co-transfected with 1.5μg pHBV 1.3 (genotype B and C) and 20nM control siRNA or siSEL1L and harvested after 72h. HBV DNA and secreted viral proteins were detected as descried above. (B and D) Huh7 cells in 12-well plates were co-transfected with 1.5μg pHBV 1.3 (genotype B and C) and 1.5μg vector or pSEL1L. After 72h, HBV DNA and secreted viral proteins were detected as descried above.

**1.3 Supplementary Figure 3**


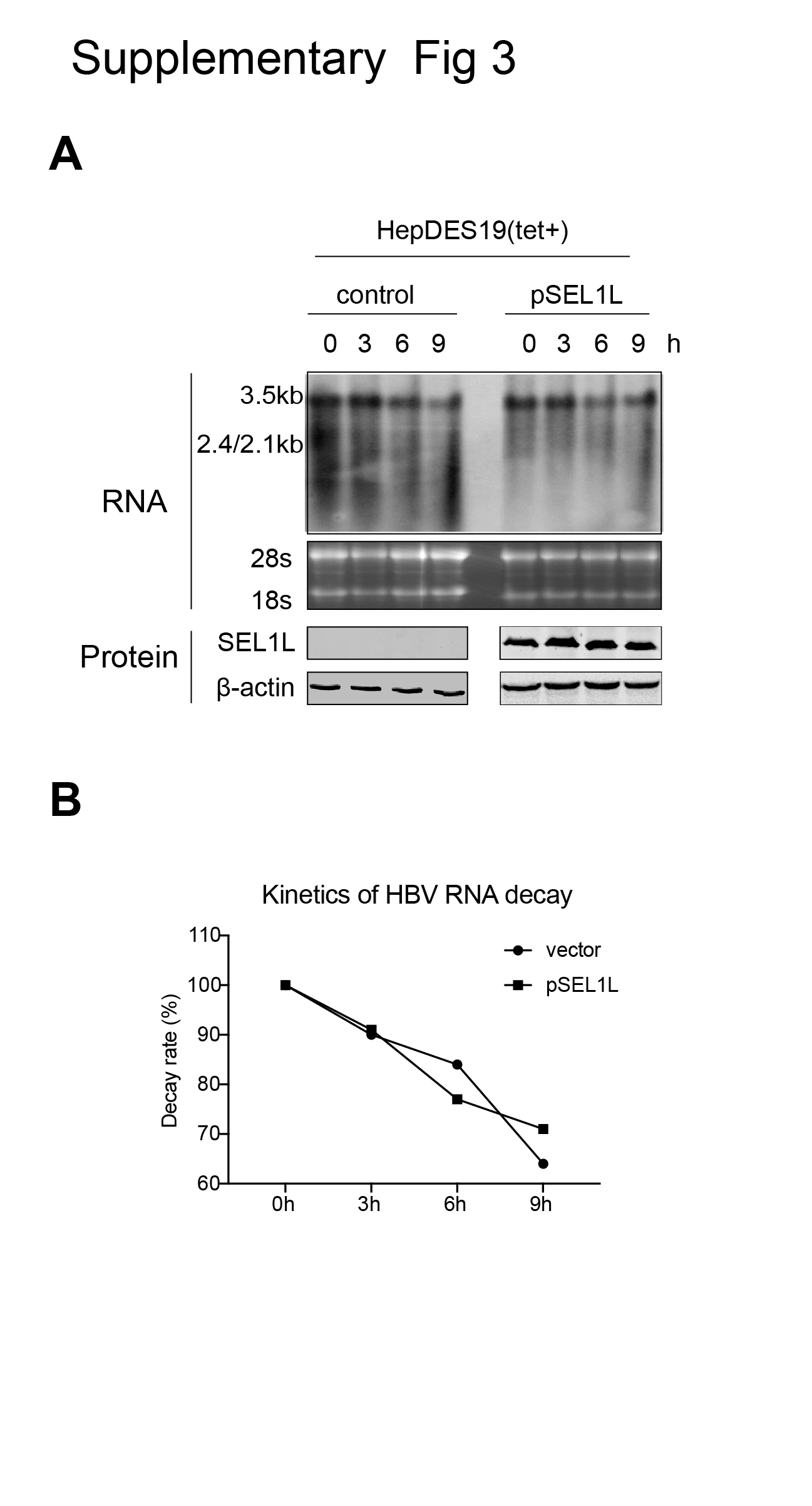


**Supplementary Figure 3.** (A) HepDES19 cells were seeded in 35 mm-dish and cultured with tetracycline-free medium to induce HBV RNA expression. One day later, cells were transfected with 4 mg of control vector or plasmid SEL1L for 36 h, then tetracycline was added back to the culture medium to shut down pgRNA transcription. Cells were harvested at indicated time points. HBV RNA was extracted from harvested samples and analyzed by Northern blot. Expression of FLAG-tagged SEL1L was detected by Western blot. (B) Kinetics analysis of HBV RNA decay in the absence or presence of SEL1L overexpression. The relative levels of HBV RNA from each sample were expressed as the percentage of the RNA signals from the corresponding sample at time point 0 h.

**1.4 Supplementary Figure 4**


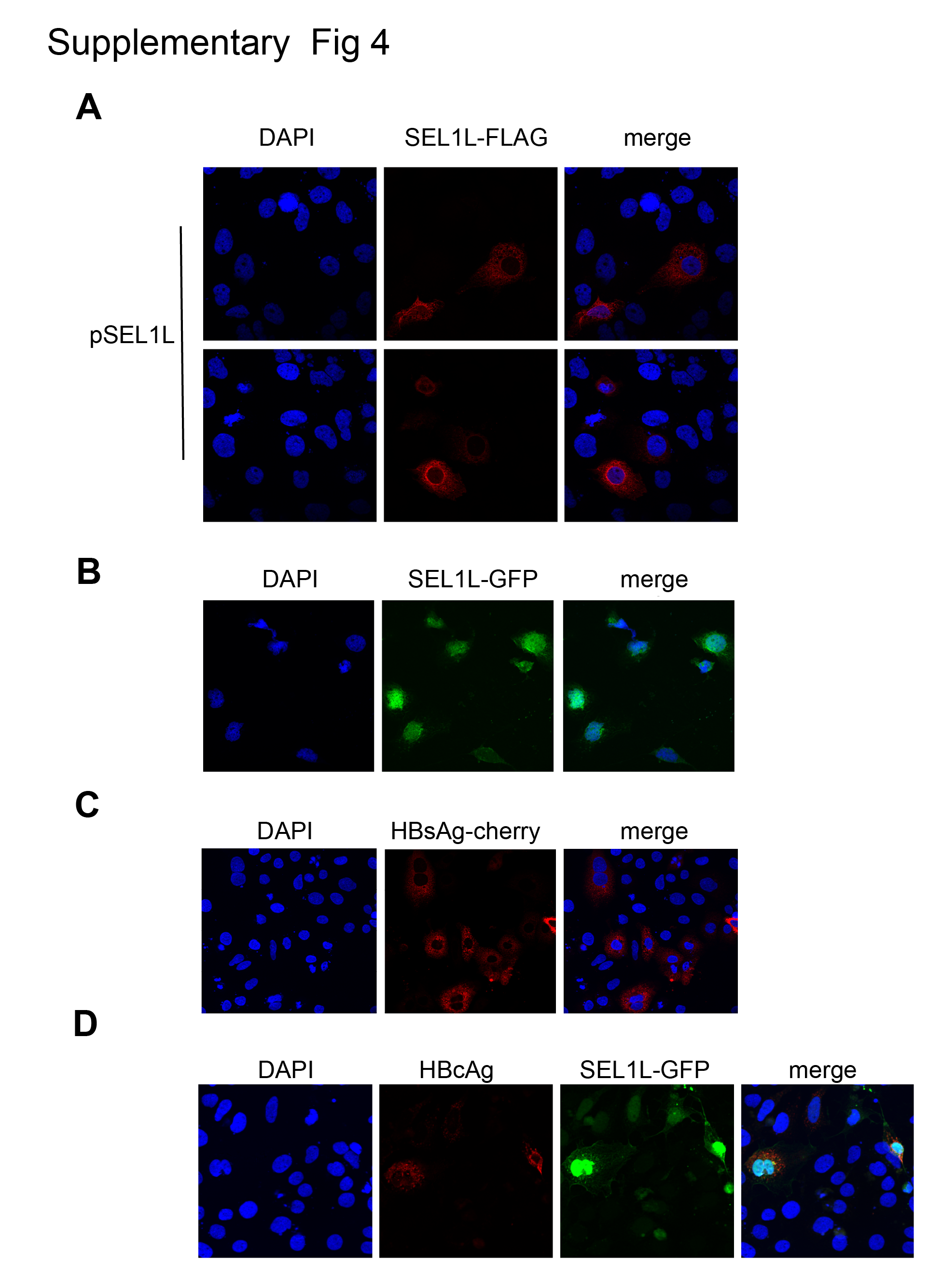


**Supplementary Figure 4.** (A) SEL1L located in cytoplasm. Huh7 cells in 12-well plates were transfected with 1.5μg pSEL1L plasmid. The cells were harvested at 48h post transfection and fixed and stained with 6-diamidino-2-phenylindole (DAPI, blue) and anti-SEL1L antibody (red). (B) The intracellular location of SEL1L-GFP. Huh7 cells in 12-well plates were transfected with 1.5μg pSEL1L-GFP plasmid. The cells were harvested at 48h post transfection and fixed and stained with second antibody. (C) The intracellular location of HBsAg-cherry. Huh7 cells in 12-well plates were transfected with 1.5μg HBsAg-cherry plasmid. The cells were harvested at 48h post transfection and fixed and stained with second antibody.(D) The co-localization of HBcAg and SEL1L. Huh7 cells in 12-well plates were co-transfected with 1.5μg pHBV 1.3 and SEL1L-GFP. The cells were harvested at 48h post transfection and fixed and stained with second antibody.

# Supplementary Tables

## Supplementary Table 1

**Table 1 Clinical and virological features of CHB patients in the study**

|  | Immune Tolerant (IT,n=22) | Immune Active (IA,n=36) | Inactive Carrier(IC,n=11) | HBeAg-Negative Hepatitis (ENH, n=14) |
| --- | --- | --- | --- | --- |
| Age (yrs)* | 30(23-33) | 41(21-65) | 42(35-50) | 45(33-72) |
| Gender (M/F) | 14/8 | 26/10 | 9/2 | 10/4 |
| HBV DNA titer** | 7.74(7.23-9.32) | 6.75(3.10-8.69) | 2.71(2.0-3.12) | 5.43(2.69-6.86) |
| HBsAg(+/-) | 22/0 | 36/0 | 11/0 | 14/0 |
| HBeAg(+/-) | 22/0 | 36/0 | 0/11 | 0/14 |
| ALT (U/L)* | 30 (13-35) | 156(59-1554) | 23(7-44) | 110(46-406) |
| AST (U/L)* | 23 (11-35) | 83(38-706) | 22(10-33) | 72(34-217) |
| Scheuer-G score(0-1/2-4) | 22/0 | 10/26 | 11/0 | 3/11 |
| 0 | 12 | 1 | 10 | 0 |
| 1 | 10 | 9 | 1 | 3 |
| 2 | 0 | 15 | 0 | 6 |
| 3 | 0 | 9 | 0 | 3 |
| 4 | 0 | 2 | 0 | 2 |
| Scheuer-S score(0-1/2-4) | 20/2 | 11/25 | 10/1 | 3/11 |
| 0 | 16 | 5 | 7 | 1 |
| 1 | 4 | 6 | 3 | 2 |
| 2 | 2 | 11 | 1 | 8 |
| 3 | 0 | 9 | 0 | 2 |
| 4 | 0 | 5 | 0 | 1 |
| Genotype(B/C) | 10/12 | 20/16 | 4/7 | 9/6 |

IT: immune tolerant, IA: immune active, IC: inactive carrier state, ENH: HBeAg-negative hepatitis.

* expressed as X±SD; **log10 copies/ml.
